# Genetically predicted susceptibility to dust-induced lung diseases and risk of autoimmune diseases: a two sample Mendelian randomization study

**DOI:** 10.1186/s12974-025-03655-5

**Published:** 2026-01-10

**Authors:** Youjin Kim, Maiko Hajime Sumikawa, Wanhyung Lee, Seunghyun Lee

**Affiliations:** 1 Asan medical center, Seoul, Republic of Korea; 2 The First Department of Internal Medicine, School of Medicine, University of Occupational and Environmental Health, Kitakyushu, Japan; 3 Department of Preventive Medicine, College of Medicine, Chung-Ang University, Seoul, Republic of Korea; 4 Department of Convergence Medicine, School of Medicine, Pusan National University, 49, Busandaehak-ro, Mulgeum-eup, Yangsan, Gyeongsangnam-do Republic of Korea

**Keywords:** Mendelian randomization, Dust-related lung diseases, Dust exposure, Lung diseases due to external agents, External airborne agent, Autoimmune diseases, Inflammation

## Abstract

**Background:**

Observational studies have linked occupational and environmental dust exposure to increased risk of autoimmune diseases (AIDs). However, it remains unclear whether genetic susceptibility to dust-induced lung pathology has a causal effect on AID risk. This study aimed to determine whether genetic susceptibility to dust-induced lung diseases causally influences AIDs risk using Mendelian randomization (MR).

**Methods:**

We conducted a two-sample MR analysis using genetic variants associated with lung diseases due to external agents (ICD-10 J60-J70; FinnGen, *n* = 500,348), and AIDs (UK Biobank, *n* = 53,831). Analyses included inverse variance weighting (IVW), MR-Egger, and weighted median methods, complemented by sensitivity analyses for heterogeneity and pleiotropy. Bonferroni and false discovery rate (FDR) corrections were applied to account for multiple testing.

**Results:**

Genetically predicted susceptibility to dust-induced lung diseases showed largely null effects for most AIDs. A suggestive association was observed for ankylosing spondylitis (AS) in the primary analysis (IVW OR 1.39, 95% CI 1.05–1.84), but became non-significant after Bonferroni and FDR corrections. Sensitivity analyses did not reveal strong evidence of horizontal pleiotropy. Thus, while a potential signal exists for AS, no robust causal effects were identified for other AIDs.

**Conclusions:**

In our study, susceptibility to dust-induced lung diseases was not robustly associated with most AIDs, but showed a suggestive disease-specific signal for AS, plausibly mediated by lung inflammation and remodeling. Other AIDs may rely on alternative systemic pathways independent of overt lung damage. Our findings highlight the mechanistic heterogeneity in dust-related autoimmunity and should be interpreted as hypothesis-generating, warranting validation in independent, larger cohorts.

**Supplementary Information:**

The online version contains supplementary material available at 10.1186/s12974-025-03655-5.

## **Introduction**

Exposure to occupational and environmental dust has been widely recognized as a significant risk factor contributing to respiratory conditions through persistent inflammatory and immune-mediated responses [1–3]. Chronic inhalation of mineral, metal, or organic dust particles such as silica, coal, or grain dust elicits oxidative stress, macrophage activation, and cytokine release in the lung parenchyma, which can extend beyond the lung to provoke systemic inflammatory responses [4]. Epidemiological and clinical studies have shown that dust-induced lung diseases, including pneumoconiosis, silicosis, and hypersensitivity pneumonitis, are associated with an increased risk of autoimmune diseases (AIDs) such as rheumatoid arthritis, systemic sclerosis, and systemic lupus erythematosus [5–7]. However, traditional observational studies are limited by confounding, measurement error, and reverse causation, making causal inference uncertain.

Mendelian randomization (MR) is a valuable approach by using genetic variants as instrumental variables (IVs) to infer causality while minimizing such biases [8]. Although dust exposure and AIDs may be mechanistically linked through dust-induced lung disease, the underlying pathway remains uncertain. Building on epidemiological evidence, we hypothesized that inherited variation in immunoinflammatory responses to chronic dust inhalation, captured through genetic variants associated with lung diseases due to external agents (ICD-10 codes J60 to J70), may causally influence AIDs risk [9]. To test this mechanistic hypothesis, we conducted a two-sample MR analysis using these genetic variants as instrumental variables [10].

This study therefore aimed to investigate whether genetically predicted susceptibility to dust-induced lung diseases causally influences the risk of AIDs. This approach allows us to test whether individuals genetically predisposed to dust-related lung injury also have a higher risk of developing AIDs, independent of behavioral or environmental confounders. By integrating large-scale genome-wide association studies (GWAS) data from the FinnGen and UK Biobank cohorts, this analysis provides novel genetic evidence for the potential role of chronic dust-related inflammatory responses in the pathogenesis of autoimmune disease, thereby bridging environmental epidemiology and genetic causal inference.

## Methods

### Study design

**Fig. 1:**
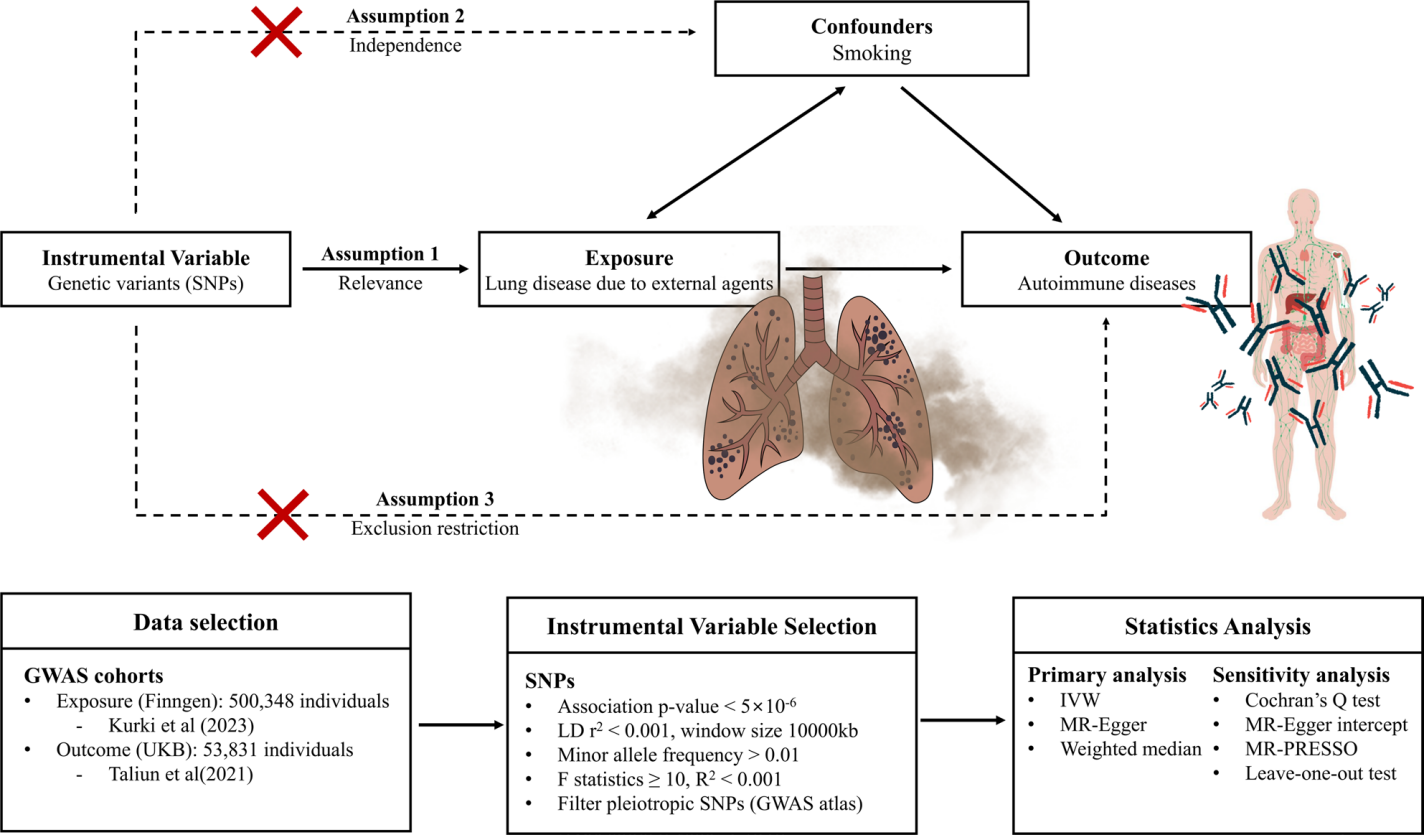
Overview of this Mendelian randomization study

The main framework for current study demonstrated in Fig. 1. We conducted a two-sample MR analysis between lung diseases due to external agents and AIDs using publicly available summary-level statistics. Three IV assumptions were satisfied to implement MR. First, genetic variants were associated with exposure (relevance assumption). Second, the variants do not share unmeasured causes with the outcome (the independence assumption). Third, the variants had no effect on the outcome except for their potential effect on exposure (exclusion restriction) [11]. All analyses were completed using R version 4.4.3 (R Core Team, 2024, R Foundation for Statistical Computing, Vienna, Austria). The R package “Two Sample MR” version 0.6.21 was used for the overall process [12, 13] and “MRPRESSO” version 1.0 for sensitivity analysis [14].

### Data source

Lung diseases due to external agents (ICD-10 codes J60–J70) were defined according to the WHO classification system [9], which is also applied in the FinnGen database. In FinnGen, lung diseases due to external agents includes pneumoconiosis, airway disease due to specific organic dust, hypersensitivity pneumonitis due to organic dust, and other respiratory conditions resulting from the inhalation of chemicals, gases, fumes, vapours, or other external agents. The lung diseases due to external agents category largely encompasses dust-related lung conditions such as pneumoconiosis and hypersensitivity pneumonitis, consistent with prior genetic and epidemiologic studies identifying associations between dust exposure and respiratory pathology [15, 16]. Although this category also includes pneumonitis due to solids and liquids (J69), separate summary statistics are not available. However, when we checked for instrument overlap with J69, no genome-wide suggestive associations were detected, suggesting that the selected IV mainly capture dust-related pathology. We used independent dataset from European population. Exposure data, ‘Lung diseases due to external agents’ which consists of 5,753 cases and 494,595 controls, were obtained from FinnGen, and accessed via https://r12.finngen.fi/ [17]. FinnGen is a public–private research initiative that integrates genotype data from Finnish biobanks with national health registry data to advance understanding of disease genetics. We used data derived from Taliun et al. [18] for outcome data. It analyzed genome-wide associations of phecode among white British participants of the UK biobank (UKB). The phecode was derived from the International Classification of Diseases codes in the electronic health records. AIDs data used as outcome were selected based on previous studies [6, 19]. All data were downloaded from https://pheweb.org/UKB-TOPMed/. As the original files have some missing reference single nucleotide polymorphism (SNP)-cluster identification information, we imputed them using the R package “SNPlocs.Hsapiens.dbSNP155.GRCh38” version 0.99.24 from Bioconductor, by comparing chromosome and base pair locations. For confounder smoking, GWAS catalog (https://www.ebi.ac.uk/gwas/) was used to find associations.

### Genetic instrument selection

We extracted genetic instruments for lung diseases due to external agents that reached genome-wide significance (p-value < 5 × 10⁻⁶) and were not associated with confounders. SNPs in linkage disequilibrium (LD) with r^2^ < 0.001 within a 10,000 kb window and SNPs harboring HLA region (chromosome 6: 25 Mb to37Mb) were excluded to ensure independence [20]. LD reference panels for each population were made using data from 1000 Genomes project [21, 22]. Variants that were located on the sex chromosome, non-biallelic, non-SNP, and had a minor allele frequency < 0.01 were excluded. Additionally, the R^2^ and F-statistics were calculated by removing SNPs with F < 10 and R^2^ < 0.001, as they were considered weak instruments. After data harmonization, palindromic SNPs with intermediate allele frequencies were removed. The remaining SNPs were included in MR analysis. The instrument strength metrics, including F-statistics and R² values for all exposure SNPs, are provided in Supplementary Table 1 for reference.

### MR analysis

We performed inverse variance weighting (IVW) and MR-Egger and weighted the medians. IVW combines the effects of different IV [23] but is susceptible to pleiotropic bias. To address this, we additionally used MR-Egger, which accounts for direct pleiotropy [24] and the weighted median, which provides a consistent effect even if up to 50% of the IVs are invalid [25]. All results were converted into odds ratios (OR).

Sensitivity analyses were performed to obtain statistically significant results. The Cochran’s Q statistic was used to test for heterogeneity [25]. MR-Egger regression [24], MR-PRESSO [14], RadialMR package *version 1.2.1* [26] were used to test for pleiotropy and identify outlier SNP. Moreover, leave-on-out (LOO) analysis was performed to assess the consistency of the results [12].

To further evaluate potential horizontal pleiotropy, we systematically examined phenotype-wide associations of the selected genetic instruments using the LDtrait platform, which integrates linkage disequilibrium information with GWAS Catalog data to identify cross-trait associations [27]. Variants showing associations with multiple unrelated traits were iteratively excluded, and sensitivity analyses were performed with different sets of instruments. The causal estimates remained stable across all scenarios, and both MR-Egger and MR‐PRESSO tests indicated no evidence of residual pleiotropy, suggesting that the observed associations are unlikely to be driven by pleiotropic effect [28]. To account for multiple comparisons across AIDs outcomes, we applied both Bonferroni and false discovery rate (FDR) corrections to the p-values derived from the IVW model. Associations were considered significant at an FDR-adjusted q < 0.05. Multivariable MR analyses were further conducted to account for potential confounding by smoking-related genetic factors.

The reporting of this study follows the STROBE‑MR (Strengthening the Reporting of Observational Studies in Epidemiology using Mendelian Randomization) guidelines. The completed STROBE‑MR checklist is provided in the Supplementary Table 2.

## Results

**Fig. 2:**
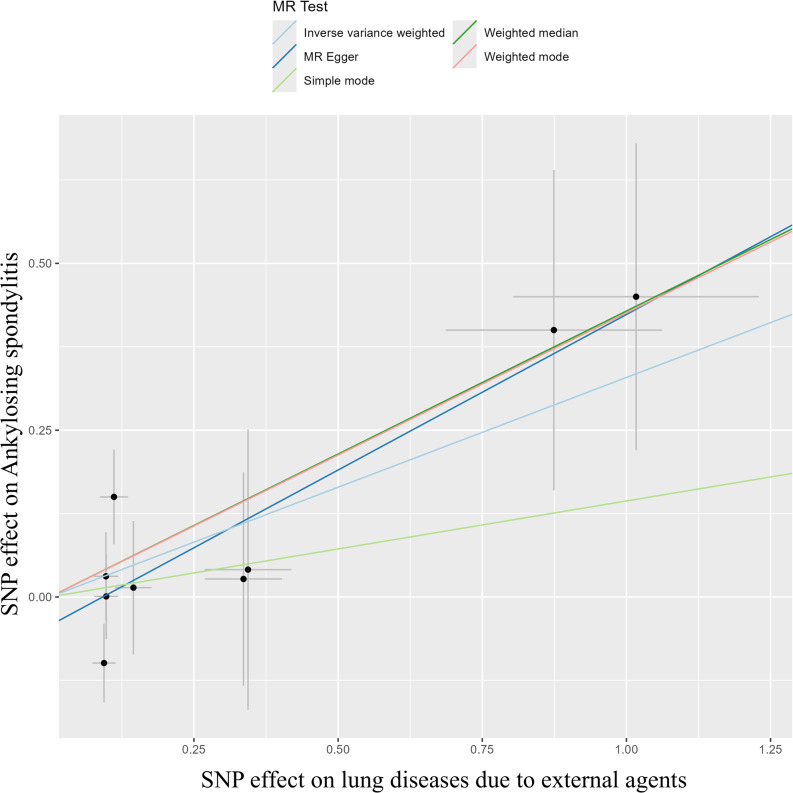
Scatter plots showing the Mendelian randomization effect of lung disease due to external agents on ankylosing spondylitis

**Fig. 3:**
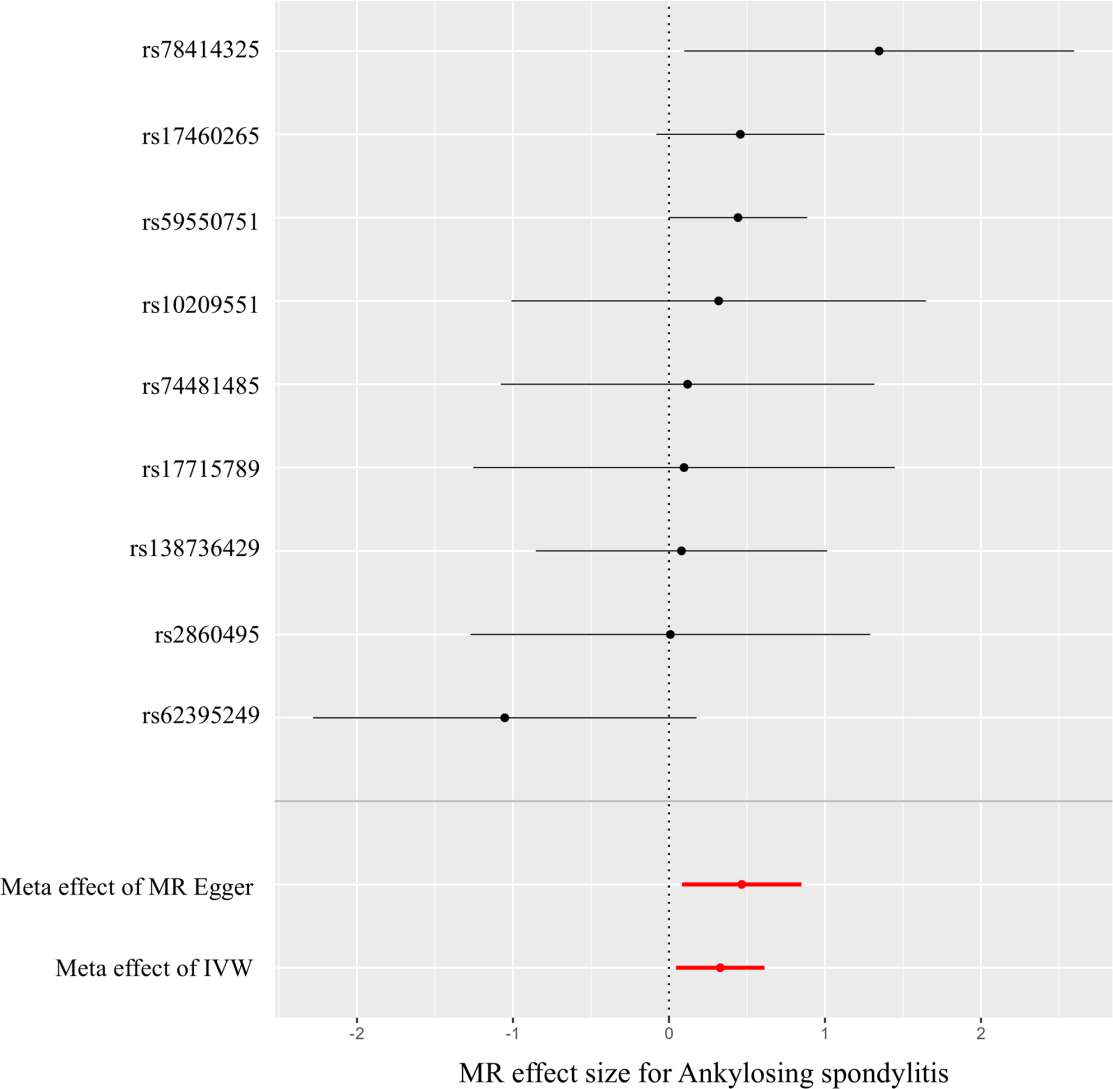
Forest plots of the Mendelian randomization effect of lung disease due to external agents on ankylosing spondylitis

We identified 10 SNPs as instrumental variables of the lung disease due to external agents, but rs564511332 were excluded since the SNP is not present in the outcome data. Thus, 9 SNPs were used for MR (Supplementary Table 1). For most AIDs, no statistically significant associations were detected. However, given the modest number of cases for certain outcomes, these null findings should be interpreted cautiously as they may partly reflect limited statistical power rather than a true absence of effect. Ankylosing spondylitis (AS) demonstrated consistent and significant associations across multiple MR methods, with odds ratios of 1.39 (95% CI: 1.05–1.84) in the IVW method, 1.59 (1.09–2.34) in MR-Egger, and 1.54 (1.03–2.28) in the weighted median analysis (Table 1). To evaluate the robustness of these findings, we performed sensitivity analyses excluding SNPs identified through LDtrait (results not shown). Excluding these variants individually and in combination produced similar estimates, indicating that horizontal pleiotropy is unlikely to account for the association between LDEA and AS. After applying Bonferroni and FDR corrections for multiple testing, no associations remained statistically significant (all *p* > 0.05). Unadjusted estimates are presented for descriptive purposes in Table 1. Figures 2 and 3 showed that the MR effect of lung disease due to external agents on ankylosing spondylitis. There were significantly closed causal effect of lung disease due to external agents on AS. All MR results related with other AIDs can be found in Supplementary Figs. 1 and 2.

**Table 1:** Mendelian randomization estimates of dust-related lung diseases on autoimmune diseases

Autoimmune diseases	Odds Ratio (95% confidence interval)		
	IVW	MR-Egger	Weighted Median
**Organ-specific diseases**			
Autoimmune hemolytic anemia	1.15 (0.57–2.31)	1.40 (0.52–3.75)	1.03 (0.42–2.52)
Pernicious anemia	1.21 (0.94–1.56)	1.30 (0.92–1.85)	1.17 (0.84–1.62)
Immune thrombocytopenia	1.09 (0.69–1.74)	1.09 (0.56–2.15)	1.34 (0.86–2.09)
Hashimoto’s thyroiditis	0.86 (0.51–1.42)	1.31 (0.70–2.45)	1.09 (0.59–2.00)
Graves’ disease	0.81 (0.59–1.11)	0.73 (0.48–1.13)	0.76 (0.50–1.17)
Type 1 diabetes	0.94 (0.81–1.10)	0.92 (0.73–1.14)	0.97 (0.80–1.16)
Primary adrenal insufficiency	1.11 (0.71–1.73)	0.86 (0.48–1.55)	1.00 (0.59–1.68)
Multiple sclerosis	1.00 (0.84–1.21)	1.08 (0.84–1.39)	1.05 (0.83–1.32)
Myasthenia gravis	1.08 (0.67–1.76)	0.85 (0.44–1.65)	0.90 (0.47–1.73)
Celiac disease	1.05 (0.90–1.23)	1.11 (0.90–1.38)	1.01 (0.82–1.24)
Crohn’s disease	1.15 (0.98–1.35)	1.18 (0.93–1.49)	1.17 (0.94–1.45)
Ulcerative colitis	1.05 (0.91–1.22)	1.17 (0.99–1.39)	1.10 (0.93–1.30)
Psoriasis vulgaris	0.91 (0.73–1.12)	0.83 (0.62–1.11)	0.94 (0.73–1.21)
Vitiligo	1.86 (0.76–4.54)	1.92 (0.53–6.95)	1.88 (0.59–5.93)
Primary biliary cholangitis	1.35 (0.85–2.12)	1.55 (0.82–2.95)	1.52 (0.88–2.65)
**Connective tissue diseases**			
Ankylosing spondylitis	**1.39 (1.05–1.84)**	**1.59 (1.09–2.34)**	**1.54 (1.03–2.28)**
Polymyalgia Rheumatica	0.91 (0.75–1.11)	0.96 (0.74–1.26)	0.95 (0.73–1.23)
Rheumatoid arthritis	1.00 (0.88–1.14)	1.06 (0.89–1.27)	1.01 (0.87–1.18)
Sarcoidosis	1.13 (0.79–1.61)	1.29 (0.79–2.12)	1.40 (0.90–2.16)
Sjögren disease	0.92 (0.68–1.25)	0.97 (0.63–1.50)	0.91 (0.62–1.34)
Systemic lupus erythematosus	0.94 (0.55–1.58)	1.58 (0.96–2.59)	1.26 (0.75–2.11)
Systemic sclerosis	1.30 (0.78–2.17)	1.15 (0.58–2.30)	1.15 (0.57–2.30)

*Bolds: *p*-value < 0.05. *IVW* Inverse Variance Weighted; *MR *Mendelian randomization

The results of the sensitivity analysis with detailed sample size are presented in Table 2. For immune thrombocytopenia (ITP), heterogeneity (*p* = 0.043) was detected, and SLE showed both heterogeneity (*p* = 0.036) and potential pleiotropy based on MR-PRESSO (*p* = 0.017). We reassessed the results after excluding outliers identified by RadialMR, and both had non-significant results. ITP, showed odds ratios of 1.20 (0.79–1.81) in IVW, 1.21 (0.66–2.20) in MR-Egger, and 1.35 (0.86–2.11) in weighted median method. For SLE, the corresponding odds ratios were 1.07 (0.73–1.55), 1.45 (0.88–2.40), and 1.28 (0.77–2.11), respectively. In additional multivariable MR analyses adjusting for smoking behavior, no significant associations were detected, indicating robustness of the main findings (results not shown).

**Table 2:** Heterogeneity and horizontal Pleiotropy tests in Mendelian randomization

Autoimmune diseases	Sample information		Cochrane’s Q		*P*_intercept_	*P*_MR−PRESSO_
	Cases	Controls	Q-value	*P*_Q_		
**Organ-specific diseases**						
Autoimmune hemolytic anemia	103	388,395	8.757	0.363	0.460	0.584
Pernicious anemia	750	388,396	8.408	0.395	0.516	0.561
Immune thrombocytopenia	418	404,524	15.961	**0.043**	0.097	0.993
Hashimoto’s thyroiditis	210	389,743	9.671	0.289	0.391	0.089
Graves’ disease	456	389,744	7.360	0.498	0.566	0.521
Type 1 diabetes	2,762	387,081	10.809	0.213	0.250	0.710
Primary adrenal insufficiency	360	403,661	12.639	0.125	0.177	0.256
Multiple sclerosis	1,339	393,433	6.074	0.639	0.723	0.407
Myasthenia gravis	192	405,102	7.217	0.513	0.590	0.333
Celiac disease	1,846	333,378	4.682	0.791	0.834	0.451
Crohn’s disease	1,737	333,378	8.167	0.417	0.468	0.769
Ulcerative colitis	3,183	333,377	11.323	0.184	0.280	0.110
Psoriasis vulgaris	1,679	396,508	12.810	0.119	0.156	0.412
Vitiligo	60	400,950	8.716	0.367	0.481	0.950
Primary biliary cholangitis	256	398,277	9.296	0.318	0.423	0.534
**Connective tissue diseases**						
Ankylosing spondylitis	617	363,561	8.616	0.376	0.476	0.334
Polymyalgia Rheumatica	1,145	406,057	4.290	0.830	0.878	0.569
Rheumatoid arthritis	4,380	363,562	12.644	0.125	0.191	0.324
Sarcoidosis	539	400,947	12.068	0.148	0.200	0.457
Sjögren disease	511	397,694	8.400	0.395	0.518	0.754
Systemic lupus erythematosus	338	399,660	16.500	**0.036**	0.060	**0.017**
Systemic sclerosis	176	397,694	5.713	0.679	0.679	0.623

*Bolds: *p*-value < 0.05

P_Q_: *P*-value from Cochran’s Q test for heterogeneity

P_intercept_: *P*-value from the MR-Egger intercept test for directional pleiotropy

P_MR−PRESSO_: *P*-value from the MR-PRESSO global test for horizontal pleiotropy

The exclusion of individual SNPs in leave-one-out tests did not materially alter the causal estimates (see Supplementary Table 3). Reverse MR analyses using genetic instruments for AIDs (*p* < 5 × 10⁻⁶) did not reveal any causal effect on lung diseases due to external agents, supporting the unidirectional relationship from dust-related susceptibility to autoimmunity. As a negative-control outcome, we analyzed hair color that is a phenotype not biologically related to dust exposure or autoimmune mechanisms and found no statistically significant associations with our genetic instruments (results not shown).

## Discussion

This study investigated whether genetic susceptibility to dust-induced lung pathology, rather than direct dust exposure, is causally linked to the risk of AIDs using MR. Given that observational studies linking dust exposure to AIDs are prone to confounding and reverse causation, a MR approach was used to strengthen causal inference. A suggestive association was observed between genetic susceptibility to dust-related lung diseases and AS in the primary MR analysis; however, this association became non-significant after Bonferroni and FDR corrections for multiple testing. No significant associations were observed for other AIDs, either before or after multiple testing correction. These results should be interpreted cautiously, as they may represent suggestive rather than definitive evidence of association. Taken together, our findings suggest that inherited susceptibility to dust-related inflammatory and fibrotic responses may specifically contribute to AS rather than representing a general causal mechanism for all AIDs.

Previous GWAS have identified multiple genetic loci associated with susceptibility to dust-induced lung diseases, suggesting that host immune and inflammatory responses play crucial roles in mediating injury following chronic dust inhalation [10, 29]. These loci are enriched in genes regulating macrophage activation, pro-inflammatory cytokines, and fibrotic remodeling which are key pathways linking pulmonary inflammation to systemic immune dysregulation [10]. Furthermore, several loci implicated in dust-related lung diseases overlap with those involved in autoimmune conditions, indicating partially shared genetic architectures between pulmonary hypersensitivity and systemic autoimmunity [30].

Despite consistent observational links between dust exposure and several AIDs [31–35], causal inference has remained uncertain, supporting the present MR approach. Environmental and occupational dust exposures, particularly to crystalline silica and coal dust, are well-established triggers of chronic lung inflammation [6]. Persistent activation of alveolar macrophages and dendritic cells in the lungs promotes the release of pro‑inflammatory cytokines, thereby creating a systemic inflammatory milieu [36].

In this context, our findings suggest that genetic susceptibility to dust-induced lung pathology might increase the risk of AS through enhanced inflammatory responses to chronic dust inhalation. However, the lack of statistical significance after multiple testing correction necessitates caution in interpreting this association. These preliminary results require validation in independent, larger studies before firm biological inferences can be drawn. While mechanistic pathways remain to be fully validated, previous studies have suggested plausible mechanisms through which dust-related pulmonary responses may influence risk of AS. One such mechanism could involve HLA-B27-induced unfolded protein response (UPR) signaling, which may amplify the IL-23/IL-17 inflammatory pathway and drive enthesis-resident T cell responses [37, 38]. Although many autoimmune diseases have been associated with specific HLA alleles and can be influenced by environmental exposures such as crystalline silica or particulate dust, AS is predominantly a cell-mediated inflammatory disease, in which chronic dust exposure could further activate the NLRP3–IL-1β pathway in pulmonary macrophages and epithelial cells, thereby promoting IL-23/Th17 polarization and reducing regulatory T cell (Treg) function [39].

In HLA-B27 carriers, this inflammation might trigger HLA-B27 misfolding and UPR, leading to excess IL-23 production by antigen-presenting cells, up-regulating the IL-23/IL-17 pathway, expanding T helper 17 (Th17) cells, and persistent Treg dysfunction [40]. Additionally, a gut–lung axis might be implicated in dust-related AS pathogenesis; chronic dust inhalation could induce gut microbiota dysbiosis, increased intestinal permeability, and bacterial antigen translocation [41, 42]. Microbial products entering the circulation stimulate innate immunity, amplify systemic inflammation, and reinforce IL-23/IL-17 activation, linking dust exposure to peripheral immune activation.

However, our MR analyses identified no significant causal effects of dust-induced lung pathology on most other AIDs, suggesting the pathogenic mechanisms linking dust exposure to autoimmunity may be heterogeneous. In particular, the suggestive AS association may reflect a mechanism involving structural lung pathology driven by NLRP3 inflammasome-mediated epithelial remodeling, whereas the relationship between dust exposure and other AIDs may rely on alternative biological pathways. In support of this notion, inhaled dust could trigger endothelial injury and systemic inflammation via the reactive oxygen species(ROS), MAPK, and NF-κB signaling pathways, even in the absence of diagnosable pneumoconiosis [43, 44]. Such systemic immune dysregulation may involve the promotion of Th1/Th17 differentiation by transition metal-containing particles and oxidative stress-induced hematopoietic dysfunction in the bone marrow [45–47]. Thus, for many AIDs, dust exposure is likely to act as a broader environmental trigger for systemic immune disruption rather than requiring specific genetic susceptibility to dust-induced structural lung damage.

Although leveraging large-scale GWAS and a biologically grounded proxy for dust-related susceptibility enabled causal inference across a broad spectrum of AIDs, this study has certain limitations. First, although MR reduces confounding, residual horizontal pleiotropy cannot be fully excluded, even after comprehensive sensitivity analyses. Second, dust exposure was assessed indirectly through genetic susceptibility to lung diseases due to external agents, rather than direct genetic proxies for actual dust exposure. This indirect approach may introduce potential misclassification or dilution of the exposure effect, as genetic variants associated with lung disease may also reflect other non-specific pulmonary processes or vulnerabilities unrelated strictly to dust exposure, thereby affecting the precision of our causal estimates. Third, the observed causal relationships may differ according to ethnicity or population characteristics; thus, caution is necessary when generalizing the findings beyond the studied populations. Fourth, this study is the lack of sex-stratified data for lung diseases due to external agents in the FinnGen database. The currently available summary statistics do not provide separate results by sex, which precludes direct assessment of potential sex-specific genetic effects. Nevertheless, most cases included in this phenotype originate from traditionally male-dominated occupational groups, such as coal and metal workers, suggesting that the underlying genetic associations may primarily reflect male susceptibility to dust-related lung pathology. Given that ankylosing spondylitis also shows a marked male predominance, this demographic pattern may partially support the biological plausibility of the observed associations. However, future studies incorporating sex-stratified genome-wide summary data are warranted to validate these findings and to better understand potential sex-specific causal pathways. Additionally, the varying sample sizes and disease prevalence, some non-significant associations may reflect limited power rather than a true absence of causal effect [48]. Future MR studies with larger case numbers are warranted to validate these null findings. Finally, AIDs are highly heterogeneous with diverse pathogenic mechanisms, and our study did not fully capture this complexity owing to limitations in the available GWAS data. Future research should explore additional genetic instruments, specific dust exposure types, and the mechanisms underlying dust-induced autoimmunity. Despite these constraints, our MR findings support dust-related pulmonary susceptibility as a plausible, disease-specific contributor to AS rather than a universal mechanism across AIDs, and underscore the importance of integrating genetic and environmental perspectives to clarify the reason dust-related immune activation may operate selectively in AS while remaining limited in other AID.

## Conclusion

In summary, this MR analysis examined the potential causal link between genetic susceptibility to dust-induced lung disease and AIDs. In primary analyses, a suggestive association was observed between dust-related lung disease susceptibility and AS; however, none of the associations across any AIDs remained statistically significant after multiple testing correction. These findings should be interpreted as exploratory and hypothesis-generating rather than providing conclusive evidence for causal relationships. The observed associations do not support clinical implementation or changes in management practices at this stage. Future studies with larger sample sizes and independent replication cohorts are essential to validate these preliminary findings and establish whether dust-induced lung disease susceptibility genuinely influences autoimmune disease risk.

## Supplementary Information

Supplementary Material 1: Figure S1.

Supplementary Material 2: Figure S2.

Supplementary Material 3: Table S1.

Supplementary Material 4: Table S2.

Supplementary Material 5: Table S3.

## Data Availability

No datasets were generated or analysed during the current study.
