# Supplementary figures and images for: Genetically predicted susceptibility to dust-induced lung diseases and risk of autoimmune diseases: a two sample Mendelian randomization study
Source: J Neuroinflammation. 2026 Jan 10;23:67. doi: 10.1186/s12974-025-03655-5 (PMC12908371; doi:10.1186/s12974-025-03655-5)

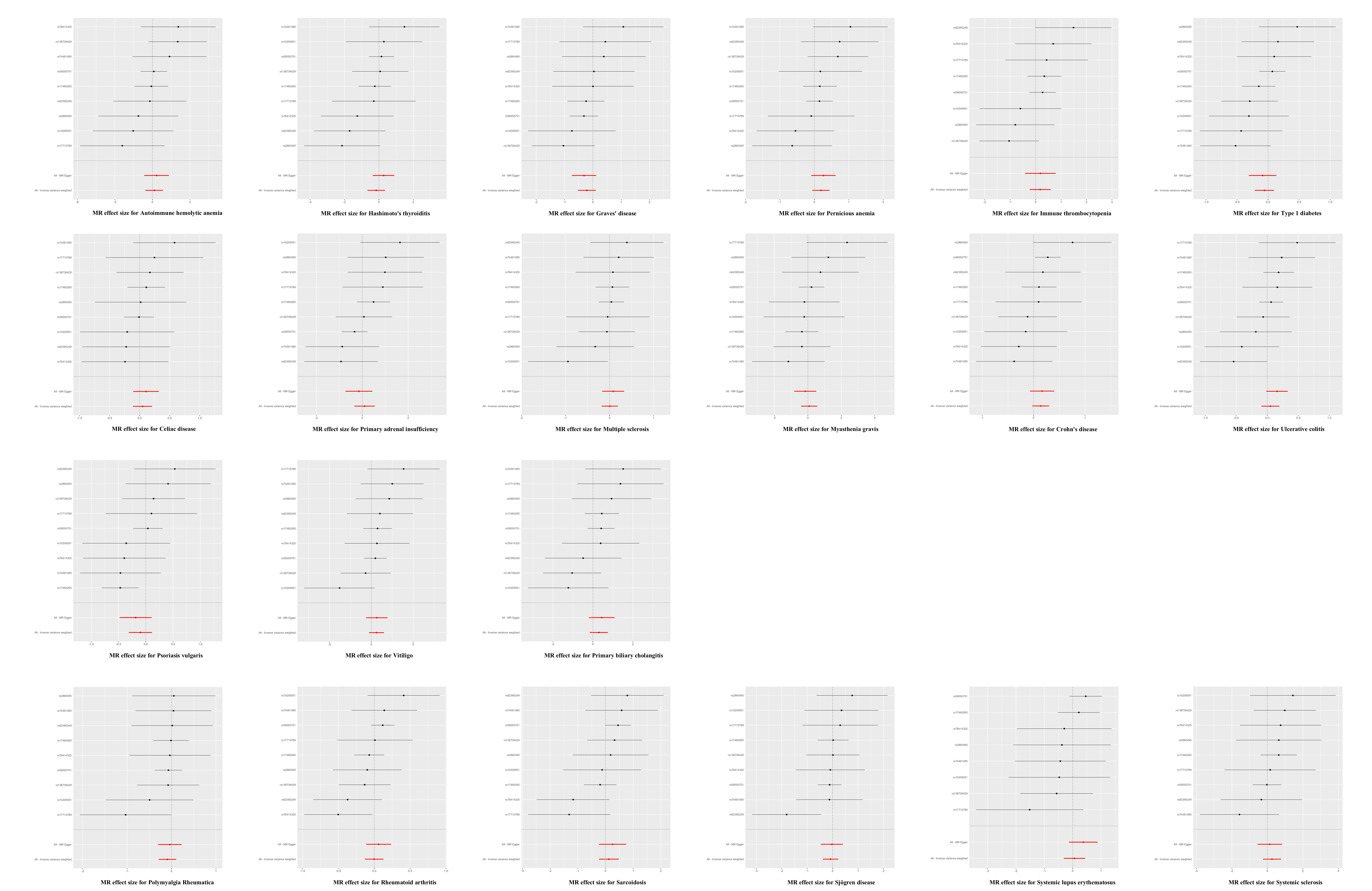

Supplement: Supplementary file 2 — Supplementary Material 2: Figure S2. [file 12974_2025_3655_MOESM2_ESM.tif]
