# Supplementary material for: Genetically predicted susceptibility to dust-induced lung diseases and risk of autoimmune diseases: a two sample Mendelian randomization study
Source: J Neuroinflammation. 2026 Jan 10;23:67. doi: 10.1186/s12974-025-03655-5 (PMC12908371; doi:10.1186/s12974-025-03655-5)
Supplement: Supplementary file 3 — Supplementary Material 3: Table S1. [file 12974_2025_3655_MOESM3_ESM.docx]

Supplementary table 1. SNP information used as instrument variables for lung disease due to external agents

| RSID | Chromosome | Position | Reference allele | Alternative allele | Nearest Gene | p-value | R² | F-statistic | Notes |
| --- | --- | --- | --- | --- | --- | --- | --- | --- | --- |
| rs10209551 | 2 | 69770863 | A | G | ANXA4 | 4.03E-06 | 4.25E-05 | 21.25 |  |
| rs59550751 | 4 | 175270775 | A | G | RP11-287F9.1 | 1.77E-06 | 4.56E-05 | 22.83 |  |
| rs62395249 | 5 | 179554365 | C | T | RUFY1 | 3.89E-06 | 4.26E-05 | 21.32 | RadialMR outlier (SLE) |
| rs138736429 | 6 | 130798763 | A | C | SMLR1 | 4.81E-07 | 5.06E-05 | 25.34 |  |
| rs74481485 | 9 | 4527103 | T | C | SLC1A1 | 4.74E-06 | 4.18E-05 | 20.94 | RadialMR outlier (ITP) |
| rs17460265 | 9 | 129288182 | G | A | RP11-344B5.2 | 3.25E-06 | 4.33E-05 | 21.66 |  |
| rs2860495 | 10 | 103515485 | T | C | NEURL1 NEURL1-AS1 | 2.52E-06 | 4.43E-05 | 22.15 |  |
| rs17715789 | 16 | 5904314 | A | G | RBFOX1 | 4.61E-06 | 4.20E-05 | 20.99 |  |
| rs78414325 | 18 | 11659404 | C | T | MIR7153 | 4.81E-06 | 4.18E-05 | 20.91 |  |

SLE: Systemic lupus erythematosus, ITP: Immune thrombocytopenia
