# Supplementary material for: Genetically predicted susceptibility to dust-induced lung diseases and risk of autoimmune diseases: a two sample Mendelian randomization study
Source: J Neuroinflammation. 2026 Jan 10;23:67. doi: 10.1186/s12974-025-03655-5 (PMC12908371; doi:10.1186/s12974-025-03655-5)
Supplement: Supplementary file 4 — Supplementary Material 4: Table S2. [file 12974_2025_3655_MOESM4_ESM.docx]

**Supplementary Table 2. STROBE-MR checklist of recommended items to address in reports of Mendelian randomization studies**^1^ ^2^

| **Item No.** | **Section** | **Checklist item** | **Page No.** | **Relevant text from manuscript** |
| --- | --- | --- | --- | --- |
| 1 | **TITLE and ABSTRACT** | Indicate Mendelian randomization (MR) as the study’s design in the title and/or the abstract if that is a main purpose of the study | 1 | “Genetically Predicted Susceptibility to Dust-induced Lung Diseases and Risk of Autoimmune Diseases: A two-sample Mendelian Randomization Study” (Title);  Abstract: “This Mendelian randomization (MR) analysis provides genetic evidence…” |
|  | **INTRODUCTION** |  |  |  |
| 2 | **Background** | Explain the scientific background and rationale for the reported study. What is the exposure? Is a potential causal relationship between exposure and outcome plausible? Justify why MR is a helpful method to address the study question | 2 | Exposure to occupational and environmental dust has been widely recognized as a significant risk factor contributing to respiratory conditions…”  “However, traditional observational studies are limited by confounding, measurement error, and reverse causation…” |
| 3 | **Objectives** | State specific objectives clearly, including pre-specified causal hypotheses (if any). State that MR is a method that, under specific assumptions, intends to estimate causal effects | 3 | “This study aimed to investigate whether genetically predicted susceptibility to dust-induced lung diseases causally influences the risk of AIDs, leveraging MR.” |
|  | **METHODS** |  |  |  |
| 4 | **Study design and data sources** | Present key elements of the study design early in the article. Consider including a table listing sources of data for all phases of the study. For each data source contributing to the analysis, describe the following: |  |  |
|  | a) | Setting: Describe the study design and the underlying population, if possible. Describe the setting, locations, and relevant dates, including periods of recruitment, exposure, follow-up, and data collection, when available. | 4-5 | Two-sample MR design using FinnGen R10 and UK Biobank GWAS data |
|  | b) | Participants: Give the eligibility criteria, and the sources and methods of selection of participants. Report the sample size, and whether any power or sample size calculations were carried out prior to the main analysis | 4-5 | ~500 000 FinnGen + > 400 000 UK Biobank participants; inclusion based on available GWAS summary statistics; no new recruitment |
|  | c) | Describe measurement, quality control and selection of genetic variants | 5 | SNPs meeting p < 5 × 10⁻⁶, r² < 0.001; harmonization, exclusion of palindromic variants, F > 10 |
|  | d) | For each exposure, outcome, and other relevant variables, describe methods of assessment and diagnostic criteria for diseases | 5-6 | Exposure = lung diseases due to external agents (ICD-10 J60–J70); outcomes = eight AIDs; diagnostic codes and GWAS case definitions specified |
|  | e) | Provide details of ethics committee approval and participant informed consent, if relevant | 6 | Publicly available summary-level data; prior ethics and consent obtained by FinnGen/UK Biobank; no new approval required |
| 5 | **Assumptions** | Explicitly state the three core IV assumptions for the main analysis (relevance, independence and exclusion restriction) as well assumptions for any additional or sensitivity analysis | 4 | “Three IV assumptions were satisfied to implement MR: relevance, independence, and exclusion restriction |
| 6 | **Statistical methods: main analysis** | Describe statistical methods and statistics used |  |  |
|  | a) | Describe how quantitative variables were handled in the analyses (i.e., scale, units, model) | 6-7 | β coefficients and SEs from GWAS used on the log-odds scale; causal estimates derived by Wald ratio and pooled with IVW random-effects model |
|  | b) | Describe how genetic variants were handled in the analyses and, if applicable, how their weights were selected | 6-7 | Independent SNPs (p < 5×10⁻⁶, r² < 0.001, 10 Mb window) selected; harmonized alleles; variants weighted by exposure β; F-stat > 10 threshold |
|  | c) | Describe the MR estimator (e.g. two-stage least squares, Wald ratio) and related statistics. Detail the included covariates and, in case of two-sample MR, whether the same covariate set was used for adjustment in the two samples | 7-8 | IVW as primary estimator; MR-Egger and weighted-median as sensitivity estimators; covariates (age, sex, ancestry PCs, batch) pre-adjusted within FinnGen and UK Biobank GWAS |
|  | d) | Explain how missing data were addressed | 8 | Not applicable—only SNPs with complete exposure and outcome summary statistics were retained after harmonization |
|  | e) | If applicable, indicate how multiple testing was addressed | 8 | Bonferroni and FDR corrections applied to IVW p-values; significance at q < 0.05; unadjusted estimates shown descriptively |
| 7 | **Assessment of assumptions** | Describe any methods or prior knowledge used to assess the assumptions or justify their validity | 7 | To further evaluate potential horizontal pleiotropy, we systematically examined phenotype-wide associations of the selected genetic instruments using the LDtrait platform… MR-Egger and MR-PRESSO tests indicated no evidence of residual pleiotropy.” |
| 8 | **Sensitivity analyses and additional analyses** | Describe any sensitivity analyses or additional analyses performed (e.g. comparison of effect estimates from different approaches, independent replication, bias analytic techniques, validation of instruments, simulations) | 7-8 | “Cochran’s Q statistic, MR-PRESSO, and RadialMR were used to assess heterogeneity and outliers. Leave-one-out analysis confirmed stability of results.” |
| 9 | **Software and pre-registration** |  |  |  |
|  | a) | Name statistical software and package(s), including version and settings used | 4 | All analyses were completed using R version 4.4.3 with the TwoSampleMR (v0.6.21) and MRPRESSO (v1.0) packages.” |
|  | b) | State whether the study protocol and details were pre-registered (as well as when and where) | 8 | “The reporting of this study follows the STROBE-MR guidelines (Supplementary Table 2).” |
|  | **RESULTS** |  |  |  |
| 10 | **Descriptive data** |  |  |  |
|  | a) | Report the numbers of individuals at each stage of included studies and reasons for exclusion. Consider use of a flow diagram | 8-9 | Exposure: FinnGen (n ≈ 500,000); Outcome: UK Biobank / IEU GWAS (n = 53,831); nine SNP instruments retained after harmonization; no individual-level exclusion |
|  | b) | Report summary statistics for phenotypic exposure(s), outcome(s), and other relevant variables (e.g. means, SDs, proportions) | 9 | Case counts and sample sizes per AID reported in Table 1; β coefficients and SEs extracted from GWAS summary statistics |
|  | c) | If the data sources include meta-analyses of previous studies, provide the assessments of heterogeneity across these studies | 9-10 | Cochran’s Q and MR-PRESSO global tests showed no significant heterogeneity across SNP estimates (p > 0.05) |
|  | d) | For two-sample MR:  i.  Provide justification of the similarity of the genetic variant-exposure associations between the exposure and outcome samples  ii.  Provide information on the number of individuals who overlap between the exposure and outcome studies | 10 | Both exposure and outcome datasets comprised European-ancestry populations with harmonized genotyping and quality control, ensuring comparable SNP–exposure effects  Exposure (FinnGen) and outcome (UK Biobank) cohorts are independent; overlap negligible, minimizing weak-instrument bias |
| 11 | **Main results** |  |  |  |
|  | a) | Report the associations between genetic variant and exposure, and between genetic variant and outcome, preferably on an interpretable scale | 10-11 | SNP–exposure and SNP–outcome β and SE extracted from FinnGen and UK Biobank GWAS; harmonized by effect allele; β on log-odds scale per risk-increasing allele |
|  | b) | Report MR estimates of the relationship between exposure and outcome, and the measures of uncertainty from the MR analysis, on an interpretable scale, such as odds ratio or relative risk per SD difference | 11-12 | IVW OR = 1.39 (95 % CI 1.12–1.71, p = 0.003) for AS; MR-Egger OR = 1.59 (95 % CI 1.03–2.44); weighted-median OR = 1.54 (95 % CI 1.11–2.13); no significant associations for other AIDs; 95 % CIs and p-values reported |
|  | c) | If relevant, consider translating estimates of relative risk into absolute risk for a meaningful time period | - | Not applicable—analysis based on summary-level data without incidence or time-to-event information |
|  | d) | Consider plots to visualize results (e.g. forest plot, scatterplot of associations between genetic variants and outcome versus between genetic variants and exposure) | 12 | Forest and scatter plots showing SNP-specific and pooled estimates provided in Figure 1 and Supplementary Figure 1 |
| 12 | **Assessment of assumptions** |  |  |  |
|  | a) | Report the assessment of the validity of the assumptions | 11-12 | Relevance confirmed by genome-wide significance (p < 5 × 10⁻⁶, F > 10); independence/exclusion restriction evaluated by MR-Egger intercept, MR-PRESSO global, and leave-one-out analyses—no pleiotropy detected |
|  | b) | Report any additional statistics (e.g., assessments of heterogeneity across genetic variants, such as *I^2^*, Q statistic or E-value) | 11-12 | Cochran’s Q p > 0.05 and I² < 25 % indicated low heterogeneity; MR-PRESSO global p = 0.33; reverse MR confirmed directionality |
| 13 | **Sensitivity analyses and additional analyses** |  |  |  |
|  | a) | Report any sensitivity analyses to assess the robustness of the main results to violations of the assumptions | 11-12 | Sensitivity analyses for robustness: Cochran’s Q for heterogeneity; MR-Egger, MR-PRESSO, RadialMR for pleiotropy; leave-one-out tests; results consistent across analyses |
|  | b) | Report results from other sensitivity analyses or additional analyses | 12-13 | Additional analyses: multivariable MR adjusting for smoking-related genetic factors; results unchanged |
|  | c) | Report any assessment of direction of causal relationship (e.g., bidirectional MR) | 12 | Directionality assessment: reverse MR using AID → lung-disease pathway showed no effect; unidirectional causal direction supported |
|  | d) | When relevant, report and compare with estimates from non-MR analyses | 12-13 | Non-MR comparison: negative-control phenotype (hair color) analyzed—no association detected |
|  | e) | Consider additional plots to visualize results (e.g., leave-one-out analyses) | 12-13 | Visualization: forest/scatter plots (Figure 2–3, Supplementary Figures 1–2) and leave-one-out plot (Supplementary Table 2) |
|  | **DISCUSSION** |  |  |  |
| 14 | **Key results** | Summarize key results with reference to study objectives | 12 | Our results demonstrated a significant association between genetic susceptibility to dust-related lung diseases and AS, whereas no significant associations were observed for other AIDs.” |
| 15 | **Limitations** | Discuss limitations of the study, taking into account the validity of the IV assumptions, other sources of potential bias, and imprecision. Discuss both direction and magnitude of any potential bias and any efforts to address them | 17-18 | This study has certain limitations… residual pleiotropy cannot be excluded… indirect proxy for dust exposure… lack of sex-stratified data… limited power… heterogeneity across AIDs.” |
| 16 | **Interpretation** |  |  |  |
|  | a) | Meaning: Give a cautious overall interpretation of results in the context of their limitations and in comparison with other studies | 15-16 | Overall interpretation given cautiously: AS association observed while most AIDs were null; results discussed in context of previous GWAS/epidemiologic evidence and limitations |
|  | b) | Mechanism: Discuss underlying biological mechanisms that could drive a potential causal relationship between the investigated exposure and the outcome, and whether the gene-environment equivalence assumption is reasonable. Use causal language carefully, clarifying that IV estimates may provide causal effects only under certain assumptions | 16-17 | Underlying biology: chronic dust exposure → NLRP3–IL-1β → IL-23/IL-17 → HLA-B27 UPR activation → cell-mediated inflammation and loss of Treg function; gene–environment equivalence noted, causal inference conditional on MR assumptions |
|  | c) | Clinical relevance: Discuss whether the results have clinical or public policy relevance, and to what extent they inform effect sizes of possible interventions | 17-18 | AS-specific findings highlight occupational and environmental implications; suggest need for preventive strategies and monitoring in dust-exposed workers |
| 17 | **Generalizability** | Discuss the generalizability of the study results (a) to other populations, (b) across other exposure periods/timings, and (c) across other levels of exposure | 17 | Caution is necessary when generalizing findings beyond the studied populations due to ethnicity and occupational exposure differences.” |
|  | **OTHER INFORMATION** |  |  |  |
| 18 | **Funding** | Describe sources of funding and the role of funders in the present study and, if applicable, sources of funding for the databases and original study or studies on which the present study is based | 19 | This research was supported by Basic Science Research Program through the National Research Foundation of Korea (NRF) funded by the Ministry of Education, Science and Technology (No. RS-2025-00520480). |
| 19 | **Data and data sharing** | Provide the data used to perform all analyses or report where and how the data can be accessed, and reference these sources in the article. Provide the statistical code needed to reproduce the results in the article, or report whether the code is publicly accessible and if so, where | 8,19 | All data used were publicly available summary statistics from FinnGen and UK Biobank; URLs provided in the Methods section.” |
| 20 | **Conflicts of Interest** | All authors should declare all potential conflicts of interest | 19 | “The authors have no conflict of interest to report.” |

This checklist is copyrighted by the Equator Network under the Creative Commons Attribution 3.0 Unported (CC BY 3.0) license.

1. Skrivankova VW, Richmond RC, Woolf BAR, Yarmolinsky J, Davies NM, Swanson SA, et al. Strengthening the Reporting of Observational Studies in Epidemiology using Mendelian Randomization (STROBE-MR) Statement. JAMA. 2021;under review.

2. Skrivankova VW, Richmond RC, Woolf BAR, Davies NM, Swanson SA, VanderWeele TJ, et al. Strengthening the Reporting of Observational Studies in Epidemiology using Mendelian Randomisation (STROBE-MR): Explanation and Elaboration. BMJ. 2021;375:n2233.
