# Supplementary material for: Genetically predicted susceptibility to dust-induced lung diseases and risk of autoimmune diseases: a two sample Mendelian randomization study
Source: J Neuroinflammation. 2026 Jan 10;23:67. doi: 10.1186/s12974-025-03655-5 (PMC12908371; doi:10.1186/s12974-025-03655-5)
Supplement: Supplementary file 5 — Supplementary Material 5: Table S3. [file 12974_2025_3655_MOESM5_ESM.docx]

| Supplementary Table 3. Leave-one-out sensitivity analysis results for the MR association between dust-related lung diseases and ankylosing spondylitis. | | | | | |
| --- | --- | --- | --- | --- | --- |
| exposure | outcome | SNP | beta | standard error | P-value |
| Lung diseases due to external agents | Ankylosing_spondylitis | rs10209551 | 0.3294 | 0.1579 | 0.0370 |
| Lung diseases due to external agents | Ankylosing_spondylitis | rs138736429 | 0.3522 | 0.1588 | 0.0266 |
| Lung diseases due to external agents | Ankylosing_spondylitis | rs17460265 | 0.2844 | 0.1763 | 0.1067 |
| Lung diseases due to external agents | Ankylosing_spondylitis | rs17715789 | 0.3389 | 0.1567 | 0.0306 |
| Lung diseases due to external agents | Ankylosing_spondylitis | rs2860495 | 0.3442 | 0.1559 | 0.0272 |
| Lung diseases due to external agents | Ankylosing_spondylitis | rs59550751 | 0.2595 | 0.1915 | 0.1753 |
| Lung diseases due to external agents | Ankylosing_spondylitis | rs62395249 | 0.4006 | 0.1429 | 0.0050 |
| Lung diseases due to external agents | Ankylosing_spondylitis | rs74481485 | 0.3405 | 0.1576 | 0.0307 |
| Lung diseases due to external agents | Ankylosing_spondylitis | rs78414325 | 0.2779 | 0.1428 | 0.0516 |
| Lung diseases due to external agents | Ankylosing_spondylitis | All | 0.3290 | 0.1446 | 0.0229 |
| Lung diseases due to external agents | Autoimmune_hemolytic_anemia | rs10209551 | 0.2139 | 0.3638 | 0.5565 |
| Lung diseases due to external agents | Autoimmune_hemolytic_anemia | rs138736429 | -0.0287 | 0.3549 | 0.9354 |
| Lung diseases due to external agents | Autoimmune_hemolytic_anemia | rs17460265 | 0.2126 | 0.4343 | 0.6245 |
| Lung diseases due to external agents | Autoimmune_hemolytic_anemia | rs17715789 | 0.2422 | 0.3469 | 0.4851 |
| Lung diseases due to external agents | Autoimmune_hemolytic_anemia | rs2860495 | 0.1959 | 0.3748 | 0.6012 |
| Lung diseases due to external agents | Autoimmune_hemolytic_anemia | rs59550751 | 0.1588 | 0.4891 | 0.7455 |
| Lung diseases due to external agents | Autoimmune_hemolytic_anemia | rs62395249 | 0.1575 | 0.3895 | 0.6859 |
| Lung diseases due to external agents | Autoimmune_hemolytic_anemia | rs74481485 | 0.0734 | 0.3746 | 0.8448 |
| Lung diseases due to external agents | Autoimmune_hemolytic_anemia | rs78414325 | 0.0382 | 0.3509 | 0.9134 |
| Lung diseases due to external agents | Autoimmune_hemolytic_anemia | All | 0.1388 | 0.3558 | 0.6966 |
| Lung diseases due to external agents | Celiac_disease | rs10209551 | 0.0601 | 0.0823 | 0.4650 |
| Lung diseases due to external agents | Celiac_disease | rs138736429 | 0.0387 | 0.0840 | 0.6449 |
| Lung diseases due to external agents | Celiac_disease | rs17460265 | 0.0281 | 0.0933 | 0.7633 |
| Lung diseases due to external agents | Celiac_disease | rs17715789 | 0.0415 | 0.0822 | 0.6134 |
| Lung diseases due to external agents | Celiac_disease | rs2860495 | 0.0507 | 0.0824 | 0.5382 |
| Lung diseases due to external agents | Celiac_disease | rs59550751 | 0.0880 | 0.1038 | 0.3969 |
| Lung diseases due to external agents | Celiac_disease | rs62395249 | 0.0628 | 0.0826 | 0.4469 |
| Lung diseases due to external agents | Celiac_disease | rs74481485 | 0.0194 | 0.0828 | 0.8150 |
| Lung diseases due to external agents | Celiac_disease | rs78414325 | 0.0641 | 0.0826 | 0.4381 |
| Lung diseases due to external agents | Celiac_disease | All | 0.0494 | 0.0806 | 0.5402 |
| Lung diseases due to external agents | chronic_lymphocytic_thyroditis | rs10209551 | -0.1769 | 0.2824 | 0.5312 |
| Lung diseases due to external agents | chronic_lymphocytic_thyroditis | rs138736429 | -0.1762 | 0.2893 | 0.5424 |
| Lung diseases due to external agents | chronic_lymphocytic_thyroditis | rs17460265 | -0.1295 | 0.3194 | 0.6851 |
| Lung diseases due to external agents | chronic_lymphocytic_thyroditis | rs17715789 | -0.1511 | 0.2835 | 0.5939 |
| Lung diseases due to external agents | chronic_lymphocytic_thyroditis | rs2860495 | -0.0639 | 0.2424 | 0.7921 |
| Lung diseases due to external agents | chronic_lymphocytic_thyroditis | rs59550751 | -0.3607 | 0.3389 | 0.2871 |
| Lung diseases due to external agents | chronic_lymphocytic_thyroditis | rs62395249 | -0.0757 | 0.2507 | 0.7626 |
| Lung diseases due to external agents | chronic_lymphocytic_thyroditis | rs74481485 | -0.2449 | 0.2447 | 0.3169 |
| Lung diseases due to external agents | chronic_lymphocytic_thyroditis | rs78414325 | -0.1005 | 0.2688 | 0.7084 |
| Lung diseases due to external agents | chronic_lymphocytic_thyroditis | All | -0.1564 | 0.2605 | 0.5481 |
| Lung diseases due to external agents | Glucocorticoid_deficiency | rs10209551 | 0.0353 | 0.2144 | 0.8691 |
| Lung diseases due to external agents | Glucocorticoid_deficiency | rs138736429 | 0.1050 | 0.2533 | 0.6784 |
| Lung diseases due to external agents | Glucocorticoid_deficiency | rs17460265 | -0.0226 | 0.2617 | 0.9312 |
| Lung diseases due to external agents | Glucocorticoid_deficiency | rs17715789 | 0.0689 | 0.2394 | 0.7735 |
| Lung diseases due to external agents | Glucocorticoid_deficiency | rs2860495 | 0.0588 | 0.2358 | 0.8030 |
| Lung diseases due to external agents | Glucocorticoid_deficiency | rs59550751 | 0.3942 | 0.2602 | 0.1298 |
| Lung diseases due to external agents | Glucocorticoid_deficiency | rs62395249 | 0.1549 | 0.2318 | 0.5038 |
| Lung diseases due to external agents | Glucocorticoid_deficiency | rs74481485 | 0.1528 | 0.2334 | 0.5126 |
| Lung diseases due to external agents | Glucocorticoid_deficiency | rs78414325 | 0.0581 | 0.2364 | 0.8059 |
| Lung diseases due to external agents | Glucocorticoid_deficiency | All | 0.1025 | 0.2269 | 0.6515 |
| Lung diseases due to external agents | Graves_disease | rs10209551 | -0.1877 | 0.1659 | 0.2579 |
| Lung diseases due to external agents | Graves_disease | rs138736429 | -0.1363 | 0.1695 | 0.4213 |
| Lung diseases due to external agents | Graves_disease | rs17460265 | -0.2018 | 0.1908 | 0.2903 |
| Lung diseases due to external agents | Graves_disease | rs17715789 | -0.2372 | 0.1656 | 0.1522 |
| Lung diseases due to external agents | Graves_disease | rs2860495 | -0.2400 | 0.1663 | 0.1489 |
| Lung diseases due to external agents | Graves_disease | rs59550751 | -0.1409 | 0.2115 | 0.5054 |
| Lung diseases due to external agents | Graves_disease | rs62395249 | -0.2237 | 0.1694 | 0.1865 |
| Lung diseases due to external agents | Graves_disease | rs74481485 | -0.2786 | 0.1666 | 0.0945 |
| Lung diseases due to external agents | Graves_disease | rs78414325 | -0.2220 | 0.1697 | 0.1907 |
| Lung diseases due to external agents | Graves_disease | All | -0.2110 | 0.1624 | 0.1938 |
| Lung diseases due to external agents | Multiple_sclerosis | rs10209551 | 0.0457 | 0.0963 | 0.6346 |
| Lung diseases due to external agents | Multiple_sclerosis | rs138736429 | 0.0106 | 0.0984 | 0.9140 |
| Lung diseases due to external agents | Multiple_sclerosis | rs17460265 | -0.0137 | 0.1077 | 0.8988 |
| Lung diseases due to external agents | Multiple_sclerosis | rs17715789 | 0.0065 | 0.0961 | 0.9461 |
| Lung diseases due to external agents | Multiple_sclerosis | rs2860495 | 0.0198 | 0.0964 | 0.8371 |
| Lung diseases due to external agents | Multiple_sclerosis | rs59550751 | -0.0194 | 0.1225 | 0.8741 |
| Lung diseases due to external agents | Multiple_sclerosis | rs62395249 | -0.0155 | 0.0966 | 0.8725 |
| Lung diseases due to external agents | Multiple_sclerosis | rs74481485 | -0.0069 | 0.0969 | 0.9435 |
| Lung diseases due to external agents | Multiple_sclerosis | rs78414325 | 0.0010 | 0.0966 | 0.9917 |
| Lung diseases due to external agents | Multiple_sclerosis | All | 0.0046 | 0.0942 | 0.9614 |
| Lung diseases due to external agents | Myasthenia_gravis | rs10209551 | 0.0919 | 0.2560 | 0.7196 |
| Lung diseases due to external agents | Myasthenia_gravis | rs138736429 | 0.1187 | 0.2589 | 0.6465 |
| Lung diseases due to external agents | Myasthenia_gravis | rs17460265 | 0.2186 | 0.2850 | 0.4430 |
| Lung diseases due to external agents | Myasthenia_gravis | rs17715789 | -0.0148 | 0.2531 | 0.9535 |
| Lung diseases due to external agents | Myasthenia_gravis | rs2860495 | 0.0208 | 0.2542 | 0.9349 |
| Lung diseases due to external agents | Myasthenia_gravis | rs59550751 | -0.0108 | 0.3198 | 0.9730 |
| Lung diseases due to external agents | Myasthenia_gravis | rs62395249 | 0.0477 | 0.2537 | 0.8510 |
| Lung diseases due to external agents | Myasthenia_gravis | rs74481485 | 0.1453 | 0.2545 | 0.5679 |
| Lung diseases due to external agents | Myasthenia_gravis | rs78414325 | 0.0954 | 0.2574 | 0.7108 |
| Lung diseases due to external agents | Myasthenia_gravis | All | 0.0795 | 0.2480 | 0.7487 |
| Lung diseases due to external agents | Pernicious_anemia | rs10209551 | 0.1913 | 0.1400 | 0.1719 |
| Lung diseases due to external agents | Pernicious_anemia | rs138736429 | 0.1485 | 0.1310 | 0.2571 |
| Lung diseases due to external agents | Pernicious_anemia | rs17460265 | 0.2006 | 0.1578 | 0.2037 |
| Lung diseases due to external agents | Pernicious_anemia | rs17715789 | 0.2017 | 0.1381 | 0.1442 |
| Lung diseases due to external agents | Pernicious_anemia | rs2860495 | 0.2295 | 0.1280 | 0.0729 |
| Lung diseases due to external agents | Pernicious_anemia | rs59550751 | 0.2198 | 0.1768 | 0.2137 |
| Lung diseases due to external agents | Pernicious_anemia | rs62395249 | 0.1635 | 0.1324 | 0.2168 |
| Lung diseases due to external agents | Pernicious_anemia | rs74481485 | 0.1443 | 0.1284 | 0.2611 |
| Lung diseases due to external agents | Pernicious_anemia | rs78414325 | 0.2273 | 0.1282 | 0.0761 |
| Lung diseases due to external agents | Pernicious_anemia | All | 0.1906 | 0.1282 | 0.1372 |
| Lung diseases due to external agents | Polymyalgia_Rheumatica | rs10209551 | -0.0762 | 0.1030 | 0.4597 |
| Lung diseases due to external agents | Polymyalgia_Rheumatica | rs138736429 | -0.0943 | 0.1052 | 0.3702 |
| Lung diseases due to external agents | Polymyalgia_Rheumatica | rs17460265 | -0.1184 | 0.1158 | 0.3068 |
| Lung diseases due to external agents | Polymyalgia_Rheumatica | rs17715789 | -0.0567 | 0.1029 | 0.5818 |
| Lung diseases due to external agents | Polymyalgia_Rheumatica | rs2860495 | -0.0997 | 0.1032 | 0.3342 |
| Lung diseases due to external agents | Polymyalgia_Rheumatica | rs59550751 | -0.1084 | 0.1316 | 0.4100 |
| Lung diseases due to external agents | Polymyalgia_Rheumatica | rs62395249 | -0.0982 | 0.1034 | 0.3421 |
| Lung diseases due to external agents | Polymyalgia_Rheumatica | rs74481485 | -0.1007 | 0.1038 | 0.3319 |
| Lung diseases due to external agents | Polymyalgia_Rheumatica | rs78414325 | -0.0956 | 0.1034 | 0.3549 |
| Lung diseases due to external agents | Polymyalgia_Rheumatica | All | -0.0929 | 0.1009 | 0.3572 |
| Lung diseases due to external agents | Primary_biliary_cirrhosis | rs10209551 | 0.3676 | 0.2209 | 0.0960 |
| Lung diseases due to external agents | Primary_biliary_cirrhosis | rs138736429 | 0.4200 | 0.2257 | 0.0627 |
| Lung diseases due to external agents | Primary_biliary_cirrhosis | rs17460265 | 0.2480 | 0.2843 | 0.3831 |
| Lung diseases due to external agents | Primary_biliary_cirrhosis | rs17715789 | 0.2538 | 0.2397 | 0.2898 |
| Lung diseases due to external agents | Primary_biliary_cirrhosis | rs2860495 | 0.2667 | 0.2491 | 0.2843 |
| Lung diseases due to external agents | Primary_biliary_cirrhosis | rs59550751 | 0.2215 | 0.3163 | 0.4838 |
| Lung diseases due to external agents | Primary_biliary_cirrhosis | rs62395249 | 0.3372 | 0.2457 | 0.1699 |
| Lung diseases due to external agents | Primary_biliary_cirrhosis | rs74481485 | 0.2321 | 0.2310 | 0.3151 |
| Lung diseases due to external agents | Primary_biliary_cirrhosis | rs78414325 | 0.2924 | 0.2549 | 0.2513 |
| Lung diseases due to external agents | Primary_biliary_cirrhosis | All | 0.2969 | 0.2328 | 0.2022 |
| Lung diseases due to external agents | Primary_thromobocytopenia | rs10209551 | 0.1175 | 0.2535 | 0.6429 |
| Lung diseases due to external agents | Primary_thromobocytopenia | rs138736429 | 0.1856 | 0.2300 | 0.4197 |
| Lung diseases due to external agents | Primary_thromobocytopenia | rs17460265 | 0.0069 | 0.2845 | 0.9806 |
| Lung diseases due to external agents | Primary_thromobocytopenia | rs17715789 | 0.0727 | 0.2578 | 0.7779 |
| Lung diseases due to external agents | Primary_thromobocytopenia | rs2860495 | 0.1300 | 0.2489 | 0.6014 |
| Lung diseases due to external agents | Primary_thromobocytopenia | rs59550751 | -0.0380 | 0.3194 | 0.9053 |
| Lung diseases due to external agents | Primary_thromobocytopenia | rs62395249 | 0.0166 | 0.2298 | 0.9423 |
| Lung diseases due to external agents | Primary_thromobocytopenia | rs74481485 | 0.1813 | 0.2113 | 0.3909 |
| Lung diseases due to external agents | Primary_thromobocytopenia | rs78414325 | 0.0569 | 0.2549 | 0.8235 |
| Lung diseases due to external agents | Primary_thromobocytopenia | All | 0.0877 | 0.2375 | 0.7121 |
| Lung diseases due to external agents | Psoriasis_vulgaris | rs10209551 | -0.0873 | 0.1164 | 0.4534 |
| Lung diseases due to external agents | Psoriasis_vulgaris | rs138736429 | -0.1214 | 0.1175 | 0.3014 |
| Lung diseases due to external agents | Psoriasis_vulgaris | rs17460265 | 0.0235 | 0.0988 | 0.8123 |
| Lung diseases due to external agents | Psoriasis_vulgaris | rs17715789 | -0.1076 | 0.1171 | 0.3583 |
| Lung diseases due to external agents | Psoriasis_vulgaris | rs2860495 | -0.1237 | 0.1104 | 0.2626 |
| Lung diseases due to external agents | Psoriasis_vulgaris | rs59550751 | -0.1857 | 0.1382 | 0.1788 |
| Lung diseases due to external agents | Psoriasis_vulgaris | rs62395249 | -0.1323 | 0.1047 | 0.2064 |
| Lung diseases due to external agents | Psoriasis_vulgaris | rs74481485 | -0.0793 | 0.1142 | 0.4873 |
| Lung diseases due to external agents | Psoriasis_vulgaris | rs78414325 | -0.0838 | 0.1159 | 0.4694 |
| Lung diseases due to external agents | Psoriasis_vulgaris | All | -0.0991 | 0.1083 | 0.3602 |
| Lung diseases due to external agents | Regional_enteritis | rs10209551 | 0.1509 | 0.0882 | 0.0870 |
| Lung diseases due to external agents | Regional_enteritis | rs138736429 | 0.1611 | 0.0882 | 0.0677 |
| Lung diseases due to external agents | Regional_enteritis | rs17460265 | 0.1490 | 0.1016 | 0.1426 |
| Lung diseases due to external agents | Regional_enteritis | rs17715789 | 0.1402 | 0.0910 | 0.1234 |
| Lung diseases due to external agents | Regional_enteritis | rs2860495 | 0.1092 | 0.0847 | 0.1970 |
| Lung diseases due to external agents | Regional_enteritis | rs59550751 | 0.0401 | 0.1085 | 0.7114 |
| Lung diseases due to external agents | Regional_enteritis | rs62395249 | 0.1364 | 0.0916 | 0.1363 |
| Lung diseases due to external agents | Regional_enteritis | rs74481485 | 0.1645 | 0.0848 | 0.0522 |
| Lung diseases due to external agents | Regional_enteritis | rs78414325 | 0.1601 | 0.0848 | 0.0590 |
| Lung diseases due to external agents | Regional_enteritis | All | 0.1386 | 0.0836 | 0.0973 |
| Lung diseases due to external agents | Rheumatoid_arthritis | rs10209551 | -0.0201 | 0.0643 | 0.7552 |
| Lung diseases due to external agents | Rheumatoid_arthritis | rs138736429 | 0.0099 | 0.0724 | 0.8908 |
| Lung diseases due to external agents | Rheumatoid_arthritis | rs17460265 | 0.0204 | 0.0800 | 0.7990 |
| Lung diseases due to external agents | Rheumatoid_arthritis | rs17715789 | -0.0023 | 0.0724 | 0.9746 |
| Lung diseases due to external agents | Rheumatoid_arthritis | rs2860495 | 0.0028 | 0.0722 | 0.9691 |
| Lung diseases due to external agents | Rheumatoid_arthritis | rs59550751 | -0.0814 | 0.0782 | 0.2978 |
| Lung diseases due to external agents | Rheumatoid_arthritis | rs62395249 | 0.0163 | 0.0654 | 0.8036 |
| Lung diseases due to external agents | Rheumatoid_arthritis | rs74481485 | -0.0095 | 0.0717 | 0.8944 |
| Lung diseases due to external agents | Rheumatoid_arthritis | rs78414325 | 0.0231 | 0.0584 | 0.6925 |
| Lung diseases due to external agents | Rheumatoid_arthritis | All | -0.0018 | 0.0664 | 0.9779 |
| Lung diseases due to external agents | Sarcoidosis | rs10209551 | 0.1325 | 0.1978 | 0.5028 |
| Lung diseases due to external agents | Sarcoidosis | rs138736429 | 0.1024 | 0.2019 | 0.6120 |
| Lung diseases due to external agents | Sarcoidosis | rs17460265 | 0.2260 | 0.2099 | 0.2816 |
| Lung diseases due to external agents | Sarcoidosis | rs17715789 | 0.1785 | 0.1651 | 0.2797 |
| Lung diseases due to external agents | Sarcoidosis | rs2860495 | 0.1187 | 0.1990 | 0.5508 |
| Lung diseases due to external agents | Sarcoidosis | rs59550751 | -0.0932 | 0.2137 | 0.6627 |
| Lung diseases due to external agents | Sarcoidosis | rs62395249 | 0.0875 | 0.1907 | 0.6464 |
| Lung diseases due to external agents | Sarcoidosis | rs74481485 | 0.0980 | 0.1953 | 0.6160 |
| Lung diseases due to external agents | Sarcoidosis | rs78414325 | 0.1872 | 0.1645 | 0.2552 |
| Lung diseases due to external agents | Sarcoidosis | All | 0.1217 | 0.1819 | 0.5036 |
| Lung diseases due to external agents | Sicca_syndrome | rs10209551 | -0.0972 | 0.1654 | 0.5568 |
| Lung diseases due to external agents | Sicca_syndrome | rs138736429 | -0.0864 | 0.1721 | 0.6155 |
| Lung diseases due to external agents | Sicca_syndrome | rs17460265 | -0.1101 | 0.1880 | 0.5582 |
| Lung diseases due to external agents | Sicca_syndrome | rs17715789 | -0.0941 | 0.1662 | 0.5714 |
| Lung diseases due to external agents | Sicca_syndrome | rs2860495 | -0.1180 | 0.1545 | 0.4449 |
| Lung diseases due to external agents | Sicca_syndrome | rs59550751 | -0.0513 | 0.2146 | 0.8112 |
| Lung diseases due to external agents | Sicca_syndrome | rs62395249 | 0.0081 | 0.1547 | 0.9583 |
| Lung diseases due to external agents | Sicca_syndrome | rs74481485 | -0.0761 | 0.1697 | 0.6540 |
| Lung diseases due to external agents | Sicca_syndrome | rs78414325 | -0.0780 | 0.1695 | 0.6453 |
| Lung diseases due to external agents | Sicca_syndrome | All | -0.0786 | 0.1547 | 0.6115 |
| Lung diseases due to external agents | Systemic_lupus_erythematosus | rs10209551 | -0.0488 | 0.2911 | 0.8668 |
| Lung diseases due to external agents | Systemic_lupus_erythematosus | rs138736429 | -0.0223 | 0.2934 | 0.9394 |
| Lung diseases due to external agents | Systemic_lupus_erythematosus | rs17460265 | -0.1585 | 0.3224 | 0.6230 |
| Lung diseases due to external agents | Systemic_lupus_erythematosus | rs17715789 | -0.0101 | 0.2707 | 0.9703 |
| Lung diseases due to external agents | Systemic_lupus_erythematosus | rs2860495 | -0.0517 | 0.2923 | 0.8595 |
| Lung diseases due to external agents | Systemic_lupus_erythematosus | rs59550751 | -0.4200 | 0.3044 | 0.1676 |
| Lung diseases due to external agents | Systemic_lupus_erythematosus | rs62395249 | 0.0648 | 0.1914 | 0.7351 |
| Lung diseases due to external agents | Systemic_lupus_erythematosus | rs74481485 | -0.0460 | 0.2926 | 0.8752 |
| Lung diseases due to external agents | Systemic_lupus_erythematosus | rs78414325 | -0.0549 | 0.2932 | 0.8514 |
| Lung diseases due to external agents | Systemic_lupus_erythematosus | All | -0.0665 | 0.2682 | 0.8042 |
| Lung diseases due to external agents | Systemic_sclerosis | rs10209551 | 0.2106 | 0.2652 | 0.4272 |
| Lung diseases due to external agents | Systemic_sclerosis | rs138736429 | 0.1988 | 0.2709 | 0.4630 |
| Lung diseases due to external agents | Systemic_sclerosis | rs17460265 | 0.1444 | 0.2980 | 0.6279 |
| Lung diseases due to external agents | Systemic_sclerosis | rs17715789 | 0.2692 | 0.2645 | 0.3088 |
| Lung diseases due to external agents | Systemic_sclerosis | rs2860495 | 0.2472 | 0.2653 | 0.3515 |
| Lung diseases due to external agents | Systemic_sclerosis | rs59550751 | 0.4537 | 0.3331 | 0.1731 |
| Lung diseases due to external agents | Systemic_sclerosis | rs62395249 | 0.2962 | 0.2659 | 0.2653 |
| Lung diseases due to external agents | Systemic_sclerosis | rs74481485 | 0.3661 | 0.2663 | 0.1693 |
| Lung diseases due to external agents | Systemic_sclerosis | rs78414325 | 0.2400 | 0.2659 | 0.3668 |
| Lung diseases due to external agents | Systemic_sclerosis | All | 0.2649 | 0.2593 | 0.3069 |
| Lung diseases due to external agents | Type_1_DM | rs10209551 | -0.0473 | 0.0834 | 0.5702 |
| Lung diseases due to external agents | Type_1_DM | rs138736429 | -0.0365 | 0.0831 | 0.6605 |
| Lung diseases due to external agents | Type_1_DM | rs17460265 | -0.0295 | 0.0939 | 0.7537 |
| Lung diseases due to external agents | Type_1_DM | rs17715789 | -0.0427 | 0.0804 | 0.5956 |
| Lung diseases due to external agents | Type_1_DM | rs2860495 | -0.0836 | 0.0735 | 0.2552 |
| Lung diseases due to external agents | Type_1_DM | rs59550751 | -0.1398 | 0.0956 | 0.1436 |
| Lung diseases due to external agents | Type_1_DM | rs62395249 | -0.0702 | 0.0840 | 0.4030 |
| Lung diseases due to external agents | Type_1_DM | rs74481485 | -0.0318 | 0.0748 | 0.6704 |
| Lung diseases due to external agents | Type_1_DM | rs78414325 | -0.0665 | 0.0850 | 0.4343 |
| Lung diseases due to external agents | Type_1_DM | All | -0.0584 | 0.0786 | 0.4575 |
| Lung diseases due to external agents | Ulcerative_colitis | rs10209551 | 0.0697 | 0.0720 | 0.3332 |
| Lung diseases due to external agents | Ulcerative_colitis | rs138736429 | 0.0604 | 0.0815 | 0.4585 |
| Lung diseases due to external agents | Ulcerative_colitis | rs17460265 | 0.0070 | 0.0849 | 0.9341 |
| Lung diseases due to external agents | Ulcerative_colitis | rs17715789 | 0.0328 | 0.0735 | 0.6559 |
| Lung diseases due to external agents | Ulcerative_colitis | rs2860495 | 0.0609 | 0.0786 | 0.4385 |
| Lung diseases due to external agents | Ulcerative_colitis | rs59550751 | 0.0421 | 0.1021 | 0.6798 |
| Lung diseases due to external agents | Ulcerative_colitis | rs62395249 | 0.0817 | 0.0639 | 0.2008 |
| Lung diseases due to external agents | Ulcerative_colitis | rs74481485 | 0.0401 | 0.0796 | 0.6143 |
| Lung diseases due to external agents | Ulcerative_colitis | rs78414325 | 0.0446 | 0.0805 | 0.5797 |
| Lung diseases due to external agents | Ulcerative_colitis | All | 0.0501 | 0.0740 | 0.4988 |
| Lung diseases due to external agents | Vitiligo | rs10209551 | 0.8097 | 0.4450 | 0.0688 |
| Lung diseases due to external agents | Vitiligo | rs138736429 | 0.7389 | 0.4836 | 0.1265 |
| Lung diseases due to external agents | Vitiligo | rs17460265 | 0.5820 | 0.5587 | 0.2976 |
| Lung diseases due to external agents | Vitiligo | rs17715789 | 0.4900 | 0.4445 | 0.2704 |
| Lung diseases due to external agents | Vitiligo | rs2860495 | 0.5489 | 0.4808 | 0.2536 |
| Lung diseases due to external agents | Vitiligo | rs59550751 | 0.7149 | 0.6257 | 0.2532 |
| Lung diseases due to external agents | Vitiligo | rs62395249 | 0.6015 | 0.4968 | 0.2259 |
| Lung diseases due to external agents | Vitiligo | rs74481485 | 0.5194 | 0.4694 | 0.2686 |
| Lung diseases due to external agents | Vitiligo | rs78414325 | 0.6189 | 0.4985 | 0.2145 |
| Lung diseases due to external agents | Vitiligo | All | 0.6215 | 0.4549 | 0.1718 |
| Lung diseases due to external agents | Ankylosing_spondylitis | rs138736429 | 0.4382 | 0.1536 | 0.0043 |
| Lung diseases due to external agents | Ankylosing_spondylitis | rs17460265 | 0.3834 | 0.1727 | 0.0264 |
| Lung diseases due to external agents | Ankylosing_spondylitis | rs17715789 | 0.4189 | 0.1496 | 0.0051 |
| Lung diseases due to external agents | Ankylosing_spondylitis | rs2860495 | 0.4253 | 0.1500 | 0.0046 |
| Lung diseases due to external agents | Ankylosing_spondylitis | rs59550751 | 0.3771 | 0.1915 | 0.0489 |
| Lung diseases due to external agents | Ankylosing_spondylitis | rs74481485 | 0.4218 | 0.1505 | 0.0051 |
| Lung diseases due to external agents | Ankylosing_spondylitis | rs78414325 | 0.3522 | 0.1502 | 0.0190 |
| Lung diseases due to external agents | Ankylosing_spondylitis | All | 0.4044 | 0.1462 | 0.0057 |
